# FACTORS ASSOCIATED WITH PAIN-RELATED FUNCTIONAL INTERFERENCE IN PEOPLE WITH CHRONIC LOW BACK PAIN ENROLLED IN A PHYSICAL EXERCISE PROGRAMME: THE ROLE OF PAIN, SLEEP, AND QUALITY OF LIFE

**DOI:** 10.2340/jrm.v56.38820

**Published:** 2024-11-15

**Authors:** Marta MORENO-LIGERO, Alejandro SALAZAR, Inmaculada FAILDE, Rogelio DEL PINO, M. Carmen CORONILLA, Jose A. MORAL-MUNOZ

**Affiliations:** 1 Preventive Medicine and Public Health Area, Department of Biomedicine, Biotechnology and Public Health, University of Cadiz, Cadiz; 2 Observatory of Pain, University of Cadiz, Cadiz; 3 Biomedical Research and Innovation Institute of Cadiz (INiBICA), Cadiz; 4 Department of Statistics and Operational Research, University of Cadiz, Cadiz; 5 Rehabilitation Unit of University Hospital Puerta del Mar, Cadiz; 6 Department of Nursing and Physiotherapy, University of Cadiz, Cadiz, Spain

**Keywords:** chronic pain, disability studies, low back pain, models, biopsychosocial, physical functional performance

## Abstract

**Objective:**

To identify the factors associated with the pain-related functional interference level in people with chronic low back pain.

**Design:**

Cross-sectional.

**Subjects/Patients:**

Chronic low back pain patients.

**Methods:**

Sociodemographic data, pain intensity, pain-related functional interference, physical functioning and fitness, sleep quality, anxiety and depression, social support, and health-related quality of life were recorded. Descriptive and bivariate analyses were performed. A linear regression model was carried out to identify the factors associated with the pain-related functional interference level.

**Results:**

99 participants were involved (mean age: 54.37 SD: 12.44; women: 67.7%). 37.4%, 27.3%, and 35.4% were classified into low, moderate, and high pain-related functional interference level groups, respectively. Higher pain-related functional interference was associated with higher pain intensity (β: 0.724; *p* = 0.026), worse sleep quality (β: 0.077; *p* = 0.012), worse quality of life (physical (β: –0.539; *p* < 0.001) and mental (β: –0.289; *p* < 0.001), and lower consumption of weak opioids (β: –3.408; *p* = 0.037).

**Conclusion:**

Beyond the pain experience and intensity among people with chronic low back pain, several biopsychosocial factors associated with this condition has been identified. Furthermore, higher pain intensity, worse sleep quality, worse quality of life, and weak opioids’ consumption have been related to the pain-related functional interference of this population.

## LAY ABSTRACT

It is known that chronic low back pain is a serious public health problem. This problem can become very limiting and generate functional interference in patients. Identifying the factors that are associated with this interference is of vital importance to improve their quality of life, and this is what this article pursues. Our findings allow us to conclude that, beyond the pain experience and intensity among people with chronic low back pain, several biopsychosocial factors are associated with this condition. Specifically, higher pain intensity, worse sleep quality, worse quality of life, and consumption of weak opioids are associated with higher pain-related functional interference in this population. Thus, we highlight the importance of targeting biological, psychological, and social components related to chronic low back pain in order to improve pain-related functional interference and disability in this population.

Chronic pain (CP) is a major public health problem affecting 20% of the worldwide population (1), which involves an individual, social, and economic burden (2). Specifically, chronic low back pain (CLBP) is the leading cause of disability globally (3), being a frequent reason for health and social care service utilization (2). Additionally, this condition is associated with reduced labour productivity, and consequently large direct and indirect costs (4).

Several interacting factors, such as biological, environmental, behavioural, and societal factors, contribute to both the pain and associated disability in people with CLBP (5). Among biophysical factors, subjects of advanced age and females are more likely to experience CLBP (6). Also, it has been observed that higher levels of pain intensity could imply deficits in sensorimotor and postural control, often leading to peripheral and/or central sensitization (7). Thus, the pain threshold may be considered as a predictive factor of CLBP (8), and it seems to be related to positions in work environments (9), especially in females (10). Regarding somatic and psychological symptoms, the presence of stress, anxiety, depression, or negative beliefs about pain is highly prevalent in this population (11). Moreover, mood disorders and poor sleep quality are not only linked to developing CLBP, but also to having worse pain-related outcomes and a poorer prognosis and recovery (9). In addition, social and interpersonal relationships variability also affect pain-related outcomes: increased perceived social support has been shown to decrease pain intensity and improve overall functioning in people with pain and physical disability (12). Furthermore, it has been observed that higher levels of disability are related to lower physical activity (PA) levels, suggesting an inverse association between PA and physical disability (13). All these biopsychosocial contributors have an impact on health-related quality of life (HRQOL) in people with CLBP, which is significantly associated with different levels of pain relief and functional disability (14).

Understanding the multifactorial nature of CLBP, as well as the association between functional interference due to pain and biopsychosocial determinants related to this condition, is crucial when planning patient-centred and multidimensional approaches for this population. Therefore, the aim of the present study was to identify the factors associated with the pain-related functional interference level in people with CLBP enrolled in a physical exercise (PE) programme.

## METHODS

### Participants and study setting

For this cross-sectional study, we used baseline data from all the participants who were recruited from the Rehabilitation Unit of the University Hospital Puerta del Mar in Cadiz (Spain) between September 2021 and September 2023 and enrolled in a clinical trial within the PainReApp project (Registered on Australian New Zealand Clinical Trials Registry with ID ACTRN12621000783820) (15). Therefore, the data used for this study are part of the periodic control of the clinical trial.

### Eligibility criteria

Eligible participants were (*i*) adults ≥ 18 years old, (*ii*) diagnosed with CLBP of at least 3 months’ duration, (*iii*) who were able to perform physical exercise (PE), (*iv*) who owned a smartphone with Internet access, and (*v*) who understand and write Spanish.

Individuals were excluded from the study if (*i*) they have concomitant diseases or medical contraindications (acute illness or injury, uncontrolled metabolic diseases, or dangerous arrhythmias and malignant hypertension) that prevent the performance of the PainReApp programmed PE plan (walking-based aerobic exercise, strengthening exercises with an elastic band, and stretching exercises); or (*ii*) they performed regular–moderate intensity physical activity (PA) (30 min/day and 3 times/week). The participants were screened consecutively from the records of the Rehabilitation Unit of the University Hospital Puerta del Mar by their rehabilitation physicians according to eligibility criteria. More details of the PainReApp programmed PE plan can be found elsewhere (15).

### Sample size

In the original clinical trial, the sample size was calculated based on the minimum clinically relevant differences in the level of pain and HRQOL variables. These differences were established at 3 points on the Numeric Pain Rating Scale (NPRS) and 5 points on the Short Form-12 (SF12v1). To detect these, with 95% confidence and 80% power, 90 subjects would be required. However, in anticipation of possible dropouts from the clinical trial, the sample size was increased by 10%, to 99 patients. For this cross-sectional study, we included the 99 participants, covering a multivariate regression model with 10 covariates or less (16), as is the case for our study.

### Variables and measurement instruments

Sociodemographic, anthropometric, and clinical variables included age, gender, marital status, socioeconomic level, educational level, employment status, body mass index (BMI), comorbidities (Charlson comorbidity index (CCI) (17), pain duration (months), pain-relief treatment (World Health Organization’s Pain Ladder) and the use of alternative therapies (mindfulness, yoga, Pilates, or others). All this information was recorded by a semi-structured questionnaire.

### Pain-related measurements

*Pain intensity* was measured by an NPRS (18), ranging from “0” (no pain) to “10” (worst pain imaginable). Pain intensity was classified as mild (< 4), moderate (4–7), and severe (> 7).

To assess *pain-related functional interference,* we used the Pictorial Pain Interference Questionnaire (PPIQ) (19), a minimal language dependence instrument. It consists of 10 illustrations representing daily living tasks (walking; socializing; rising from chair; climbing stairs; carrying a parcel or moderate-sized item; reaching above shoulder height; engaging in activity outside of the home; sleeping; sports/recreation; engaging in activities with family). Each item is scored by a 5-point rating scale according to how much pain affects the ability to participate in those activities. Thus, the overall score ranges between 10 and 50, indicating high pain-related interference with functioning as the score increases.

To evaluate *Physical functioning*, 3 measurements were used:

1. For *functional mobility*, the Timed Up and Go Test (TUG) was used (20). This consists of getting up from a chair, walking 3 m at a comfortable and safe pace, turning around, walking back to the chair, and sitting down. Timing starts with the command “go” and stops when the subject sits down. A TUG score of ≥ 10 s indicates reduced physical capacity (21).
2. For *upper and low body muscular strength and endurance*, the 30-s Arm Curl Test (30ACT) and the 30-se Chair Stand Test (30CST) were used (20). The purpose of those tests is recording the arm curls or stands repetitions in 30 s.
3. *For physical fitness*, the International Physical Activity Questionnaire Short Form (IPAQ-SF) was used (22). It encompasses 7 questions related to walking, moderate-intensity activities, and vigorous-intensity activities, as well as the time spent sitting. From these questions, the total PA in the metabolic equivalent of task (MET)-min/week can be calculated to classify 3 PA levels as low, moderate, and vigorous.

### Sleep and psychosocial measurements

*To assess sleep quality,* the 12-item Medical Outcomes Study Sleep (12-MOS Sleep) was used (23). It involves 12 items, which generates 6 sub-scales and 2 summary indices (6- and 9-item indexes). However, for this study, only the sleep index “I-9” was used, whose overall score ranges from 0 (best sleep quality) to 100 (worst sleep quality).

*Anxiety* and/or *depression* were assessed using the Hospital Anxiety and Depression Scale (HADS) (24). This self-assessment scale includes 14 items divided into 2 subscales of anxiety (HADS-A) and depression (HADS-D). Each item is scored from 0 to 3, resulting in an overall subscale score between 0 and 21. The cut-off points to detect the presence of anxiety or depression states are as follows: 0–7 (normal), 8–10 (borderline), and 11 or above (pathological condition).

To evaluate *social and emotional support*, the 11-item Duke-UNK Functional Social Support Questionnaire (DUKE-UNC-DSSI) was used (25). The overall score ranges between 11 and 55, with a cut-off score of 32 to categorize the individual’s perceived social support as “low” (< 32) and “normal” (≥ 32).

*HRQOL was measured using* the SF-12v1(26), which includes 12 items, yielding 2 component summaries: Mental (MCS) and Physical (PCS), which are Studentised with a mean = 50. The global score ranges from 0 to 100, with higher scores indicating better HRQOL.

### Statistical analyses

First, a descriptive analysis was carried out. For quantitative variables, central tendency (mean) and dispersion (standard deviation [SD]) were calculated, as well as the minimum and maximum values. For categorical variables, absolute and relative frequencies were reported. Also, to check the normal distribution of continuous variables, the Kolmogorov–Smirnov test was used.

Second, the factors related to pain-related functional interference were analysed using Pearson or Spearman correlation coefficients for quantitative variables (depending on the nature of the variable), and Student’s *t*-test (2 groups) and ANOVA (3 or more groups) for qualitative variables.

A multivariate analysis was subsequently carried out to identify the factors associated with the pain-related functional interference level (dependent variable: PPIQ score). We fitted a linear regression model with sociodemographic, pain-related, physical functioning, sleep and psychosocial, and HRQOL measurements with a significant association with the PPIQ score as the independent variable. A stepwise method was used to select the final set of co-variables in the model, according to the criterion of the Wald test. The level of significance was established at α = 0.05. R-square was used for the goodness of fit.

The SPSS statistical package program (v. 29; IBM Corp, Armonk, NY, USA) was used to perform all statistical analyses.

## RESULTS

### Participant characteristics

The present study included 99 participants with CLBP, 67.7% of whom were females, and the average age was 54.37 years (SD 12.44). Detailed information on sociodemographic, anthropometric, and clinical characteristics is given in Table I. Regarding the bivariate analysis, we observed that higher PPIQ scores were associated with lower socioeconomic levels (*p* = 0.033), periods of sick leave (*p* = 0.049), and consumption of non-opioid analgesic (*p* < 0.001). Also, the PPIQ score was directly associated with the higher BMI (*p* = 0.015) (Table I).

**Table I:** Sociodemographic, anthropometric, and clinical characteristics of the sample: associations with pain-related functional interference (PPIQ)

Variables	Categories	Sample (*n* = 99) *n* (%)	Pain-related functional interference (PPIQ)	
			Spearman (rho)/Pearson (*r*) Mean (SD)	*p*-value
Sociodemographic variables				
Age (years)	Mean (SD); Min–Max	54.37 (12.44); 22-78	*r* = 0.157	0.121^a^
Gender	Male	32 (32.3)	26.31 (10.89)	0.074^c^
	Female	67 (67.7)	30.03 (8.90)	
Educational level	Any studies	9 (9.1)	31.33 (9.14)	
	Primary	23 (23.2)	30.09 (8.17)	0.228^d^
	Secondary	51 (51.5)	29.20 (10.03)	
	University	16 (16.2)	24.44 (10.42)	
Socioeconomic level (*n* = 98)	Low	8 (8.2)	37 (8.12)	
	Low–Middle	36 (36.4)	29.25 (10.08)	0.033^d^
	Middle	46 (46.5)	28.24 (9.48)	
	Middle–high	8 (8.2)	23.25 (6.02)	
Marital status	Single	18 (18.2)	27.33 (10.96)	
	Married	65 (65.7)	28.97 (9.43)	0.825^d^
	Divorced/Separated	11 (11.1)	29.09 (10.74)	
	Widowed	5 (5.1)	31.80 (7.43)	
Employment status	Unemployed	22 (22.2)	30.82 (9.67)	
	Employed	46 (46.5)	26.39 (8.86)	0.049^d^
	Retired/Early retired	23 (23.2)	29.48 (10.74)	
	Sick leave	8 (8.1)	35.50 (7.91)	
Anthropometric and clinical variables				
Body mass index (kg/m)	Mean (SD); Min-Max	29 (5.40); 16.76-46.34	*r* = 0.244	0.015^a^
Charlson Comorbidities Index	Mean (SD); Min-Max	0.51 (1.03); 0-6	0.1	0.326^b^
Comorbidity	None	88 (88.9)	28.31 (9.44)	
	Low	6 (6.0)	36.33 (8.09)	0.146^d^
	High	5 (5.1)	29 (13.82)	
Pain duration (months) (*n* = 97)	Mean (SD); Min–Max	82.26 (118.47); 4-480	Rho = 0.044	0.668^b^
Non-opioid analgesics consumption (*n* = 97)	Yes	55 (56.7)	31.30 (9.21)	< 0.001^c^
Weak opioid consumption (*n* = 97)	Yes	17 (17.5)	30.71 (9.29)	0.338^c^
Strong opioid consumption (*n* = 97)	Yes	5 (5.2)	34.40 (6.88)	0.174^c^

a Pearson correlation coefficient (*r*).

b Spearman rank correlation coefficient (rho).

c Student’s *t*-test, assuming equal variances according to the Levene test.

d ANOVA.

SD: standard deviation.

Regarding pain-related measurements, the mean of pain intensity (NPRS) was 6.67. Furthermore, participants most frequently perceived their pain as “severe” (64.3%), and 37.4%, 27.3%, and 35.4% of participants were classified at low, moderate, and high pain-related functional interference levels, respectively (Table II).

**Table II:** Pain-related, physical functioning, biopsychosocial, and health-related quality of life measurements of the sample. Associations with pain-related functional interference (PPIQ)

Variables		Categories	Sample (*n* = 99) *n* (%)	Pain-related functional interference (PPIQ)	
				Spearman (rho)/Pearson (*r*) Mean (SD)	*p*-value
Pain-related measurements					
Pain intensity (NPRS) (*n* = 98)		Mean (SD)	6.67 (1.99)	Rho = 0.454	< 0.001^b^
		Mild (1–3) Moderate (4–6) Severe (7–10)	8 (8.2) 27 (27.6) 63 (64.3)	21.75 (10.99) 24.48 (7.03) 31.86 (9.24)	< 0.001^d^
Pain-related functional interference (PPIQ)		Mean (SD)	28.83 (9.69)	-	-
		High (≥ 34) Moderate (26-33) Low (≤26)	35 (35.4) 27 (27.3) 37 (37.4)	-	-
Physical functioning measurements					
Timed Up & Go Test		Mean (SD)	8.40 (3.11)	Rho = 0.522	< 0.001^b^
30-s Arm Curl Test (*n* = 97)		Mean (SD)	11.81 (4.06)	Rho = –0.328	0.001^b^
30-s Chair Stand Test		Mean (SD)	10.0 (3.78)	Rho = -0.472	< 0.001^b^
Physical fitness (IPAQ-SF)	PA total METs	Mean (SD)	2,311.34 (2,646.80)	Rho = –0.226	0.026^b^
	PA levels	Vigorous PA Moderate PA Low PA	26 (26.3) 59 (59.6) 14 (14.1)	33.21 (13.06) 29.03 (8.61) 26.00 (9.69)	0.076^d^
Biopsychosocial measurements					
Sleep quality (12-MOS Sleep I-9)		Mean (SD)	42.68 (22.46)	*r* = 0.486	< 0.001^a^
Depression (HADS-D)		Mean (SD)	5.80 (4.08)	Rho = 0.592	< 0.001^b^
		No case (0–7) Unclear (8–10) Case (≥ 11)	67 (67.7) 17 (17.2) 15 (15.2)	25.31 (8.34) 35 (7.99) 37.53 (8.34)	< 0.001^d^
Anxiety (HADS-A)		Mean (SD)	7.49 (4.79)	Rho = 0.473	< 0.001^b^
		No case (0–7) Unclear (8–10) Case (≥ 11)	57 (57.6) 20 (20.2) 22 (22.2)	25.21 (8.39) 32.40 (10.23) 34.95 (8.30)	< 0.001^d^
Social and emotional support (DUKE-UNC-DSSI) (*n* = 97)		Mean (SD)	42.55 (9.97)	Rho = *–*0.372	< 0.001^b^
		Low perceived social support (< 32) Normal support (≥ 32)	15 (15.5) 82 (84.5)	34.87 (7.92) 27.56 (9.54)	0.006^c^
Health-related quality of life measurement					
Health related quality of life (SF-12v1)		PCS, Mean (SD)	35.45 (9.13)	Rho = –0.550	< 0.001^b^
		MCS, Mean (SD)	43.96 (13.37)	Rho = –0.502	< 0.001^b^

a Pearson correlation coefficient (r).

b Spearman rank correlation coefficient (rho).

c Student’s *t*-test, assuming equal variances according to the Levene test.

d ANOVA.

DUKE-UNC-DSSI; Duke-UNK Functional Social Support Questionnaire**;** HADS: Hospital Anxiety and Depression Scale**;** IPAQ-SF: International Physical Activity Questionnaire short-form; 12-MOS Sleep: Medical Outcomes Study; MCS: Mental Component Summary; MET: Metabolic Equivalent Task; NPRS: Numeric Pain Rating Scale; PA: physical activity; PCS: physical component summary; PPIQ: Pictorial Pain Interference Questionnaire; SF-12v1: Short-Form 12 Health Survey version 1; SD: standard deviation.

With regard to sleep quality, the study shows that the mean score of the sleep index “I-9” was 42.68, slightly higher than 40, which would be interpreted as poor sleep quality. The presence of anxiety and depression was observed in 22.2% and 15.2% of participants, respectively. Also, in terms of social support, we observed that most of participants (84.5%) reported normal social support. Finally, in relation to the HRQOL, the mean of the PCS and MCS were 35.45 and 43.96, respectively. Other characteristics of the sample are indicated in Table II.

Regarding the bivariate analysis, results showed that the PPIQ score (pain-related functional interference) was associated with higher pain intensity (*p* < 0.001), participants who reported severe pain (*p* < 0.001), more time in the execution of the TUG (*p* < 0.001), worse sleep quality (*p* = 0.486, *p* < 0.001), the presence of anxiety (*p* < 0.001) and depression (*p* < 0.001) (HADS scale), fewer repetitions in the 30ACT (*p* = 0.001) and the 30CST (*p* < 0.001), lower PA total METs (*p* = 0.026), lower HRQOL (both PCS (*p* < 0.001) and MCS (*p* < 0.001)), and less social and emotional support (*p* < 0.001) (Table II).

### Factors related to pain-related functional interference: linear regression model

Multivariate analysis of the factors associated with the pain-related functional interference revealed that higher PPIQ scores were directly associated with pain intensity and worse sleep quality, and indirectly with the physical and mental components of the HRQOL, and weak opioid consumption (Table III).

**Table III:** Factors associated with pain-related functional interference among people with CLBP: linear regression model

Factor	Pain-related functional Interference (PPIQ)		
	β	95% CI	*p*-value
Pain intensity	0.724 (0.321)	(0.087; 1.360)	0.026
Sleep quality	0.077 (0.030)	(0.018; 0.137)	0.012
PSC (HRQOL)	-0.539 (0.069)	(-0.676; –0.402)	< 0.001
MSC (HRQOL)	-0.289 (0.051)	(-0.390; 0.189)	< 0.001
Weak opioid consumption Yes No*	-3.408 (1.610)	(-6.608; –0.209)	0.037

Dependent variable: pain-related functional interference (PPIQ).

MSC: mental summary component; PPIQ: Pictorial Pain Interference Questionnaire; PSC: physical summary component; HRQOL: health-related quality of life.

* Reference category.

Specifically, an increase of 0.724 points in the level of pain-related functional interference was observed for each unit of increase in pain intensity (*p* = 0.026), and 0.077 points for each unit of increase on the sleep quality scale (i.e., worse sleep quality) (*p* = 0.012).

On the other hand, each unit of increase in the score of HRQOL was associated with a decrease of approximately half a point in pain-related functional interference in the case of PCS, and 0.289 points in the case of MCS (*p* < 0.001 in both cases).

Finally, people who consumed weak opioids presented, on average, 3.408 points less on the PPIQ scale compared with those who did not consume them (Table III).

## DISCUSSION

The present study aims to identify the relationships between functional interference due to pain and several multidimensional factors in people diagnosed with CLBP enrolled in a PE programme. We observed that several sociodemographic and anthropometric, pain-related, and biopsychosocial variables could be associated with pain-related functional interference in our study population. Furthermore, the multivariate analysis showed that higher pain-related functional interference is associated with higher pain intensity, worse sleep quality, worse HRQOL (physical and mental components), and lower consumption of weak opioids.

CLBP is one of the major causes of disability, associated with multidimensional factors (5), in which biological, environmental, psychological, and social factors influence pain experience, in a bidirectional way, and the patient-related outcomes (27). Some socioeconomic factors, such as low educational level and income, could forecast pain-related disability in CLBP population (5). Moreover, the participants with CLBP involved in this study presented as overweight on average. In that sense, the literature showed the negative effects of overweight and obesity on the risk of developing CLBP because of the relationship between the secretion of inflammatory markers by adipose tissue and pain experience (28). Regarding emotional symptoms, a slight presence of anxiety and depression symptoms was observed among the study population, which are considered as risk factors for CLBP and predictors of pain and pain-related disability (29). Indeed, these psychological factors may have more influence on disability and quality of life outcomes than on pain itself (30). Similarly, perceived social and emotional support is showed to influence the recovery from depressive symptoms as well the reduction of pain intensity and disability in people with CLBP (31). Thus, Wippert et al. (32) pointed out the protective role of higher perceived social and emotional support in this population. Regarding the physical fitness information, contrary to what we might expect from previous literature (13), our results did not show significant correlations between the PA levels and the pain-related functional interference of the study sample. This could be explained by the overestimated PA results from the IPAQ observed in some research (22), which might be attributed to social desirability and recall biases (33).

In the case of pain intensity and sleep quality, these associations were direct, indicating higher scores in PPIQ (higher pain-related functional interference level) when higher pain intensity was reported and higher score on the sleep index “I-9”. As can be found in the literature, pain intensity has a negative influence on physical function and role functioning in people with CLBP (34). Thus, our findings are in line with Mutubuki et al. (35), who observed that higher pain intensity is directly associated with higher functional interference and disability in people with CLBP. Moreover, it has been shown that initial high pain intensity increases the risk of disabling CLBP (36). Therefore, the pain threshold not only seems to be a predictive factor of CLBP (8), but also a critical determinant of disability caused by this condition. Related to this, the reciprocal relationships between pain intensity and sleep quality also influence physical functioning, showing that poor sleep quality significantly predicts poorer physical disability in people with CLBP (37). In that sense, Burgess *et al*. (38) observed that sleep disorders and impaired function were conveyed statistically not only by indirect effects of increased pain intensity associated with sleep disorders, but also by direct effects of sleep disorders on function. Similarly, Zarrabian et al. (39) also found that sleep disorders predicted disability independent of pain intensity. In that sense, emerging studies have focused on identifying the causal directions between these outcomes, suggesting that sleep disorders are stronger and more reliable predictors of exacerbations of pain and functional disability than inversely (40).

Moreover, the lower score on physical and mental components of the HRQOL, and the consumption of weak opioids, were inversely associated with the PPIQ score among the population analysed. In that sense, previous literature has pointed out this inverse relationship between CLBP and HRQOL, showing. that greater disability is contributing to experiencing poorer quality of life (34). Indeed, we observed that the means of both PCS and MCS in our study population are below the expected score in the general population (mean = 50). From another point of view, Taylor *et al*. (14) observed better perceived HRQOL among population with fewer levels of functional disability. Regarding the association between the consumption of weak opioids and pain-related functional interference, Petzke *et al*. (41) observed that opioids improve pain intensity and disability levels in people suffering from CLBP. Nevertheless, the opioid use for pain relief in CLBP seems to have short-term analgesic efficacy, and less clear efficacy in terms of functional disability (42).

The findings of this study encourage the implementation of multidimensional approaches for CLBP management targeting biological, psychological, and social factors, which could be especially beneficial for pain management and functional ability among people with CLBP. Thus, the individualization of management according to personal needs, expectations, and environment is emphasized. Furthermore, considering pain-relief medications is also a key point to target because of its interactive effects on pain outcomes among people with CLBP.

### Strengths and weaknesses of the study

Finally, some strengths and weaknesses of the present study should be noted. The main strength is that it consists of a study with primary data from people diagnosed with CLBP attending a public health institution and enrolled in a study based on a physical exercise programme. Nevertheless, the generalization of our findings to a general population with CLBP or even other CP conditions must be considered with caution. In fact, it is worth mentioning that our study population involved patients with CLBP who were able to perform PE. Also, the nature of this cross-sectional study does not allow establishment of the cause–effect relationship between associated factors and pain-related functional interference in patients with CLBP enrolled in a PE programme.

### Conclusion

This study showed that CLBP and the consequent pain-related functional interference is related to multidimensional factors, and understanding these relationships is crucial for the development of effective management and treatment approaches. Furthermore, our results suggested that pain-related functional interference of patients with CLBP enrolled in a PE programme is associated with higher pain intensity, worse sleep quality, and worse HRQOL (both physical and mental components), and with lower consumption of weak opioids. Thus, we highlighted the importance of targeting biological, psychological, and social components related to CLBP in order to improve the pain-related functional interference and disability of this population.
